# Cardiovascular risk factors and cognitive decline in older people with type 2 diabetes

**DOI:** 10.1007/s00125-015-3581-0

**Published:** 2015-04-07

**Authors:** Insa Feinkohl, Markéta Keller, Christine M. Robertson, Joanne R. Morling, Stela McLachlan, Brian M. Frier, Ian J. Deary, Mark W. J. Strachan, Jackie F. Price

**Affiliations:** Centre for Population Health Sciences, Medical School, University of Edinburgh, Teviot Place, Edinburgh, EH8 9AG Scotland UK; The Queen’s Medical Research Institute, University of Edinburgh, Edinburgh, Scotland UK; Centre for Cognitive Ageing and Cognitive Epidemiology, University of Edinburgh, Edinburgh, Scotland UK; Psychology in the School of Philosophy, Psychology and Language Sciences, University of Edinburgh, Edinburgh, Scotland UK; Metabolic Unit, Western General Hospital, Edinburgh, Scotland UK

**Keywords:** Blood glucose, Blood pressure, Cardiovascular risk, Cholesterol, Cognitive impairment, Glycaemic control, Hyperglycaemia, Older age, Smoking, Type 2 diabetes

## Abstract

**Aims/hypothesis:**

The aim of this work was to assess the role of well-established cardiovascular risk factors in the late-life cognitive decline of patients with type 2 diabetes.

**Methods:**

Data from 831 participants (aged 60–75 years) attending the 4 year follow-up of the Edinburgh Type 2 Diabetes Study (ET2DS) were used. Smoking history (pack-years), BP, HbA_1c_, plasma glucose and cholesterol were determined at baseline clinics (single time measurements) and/or from serial data recorded on a clinical management database from diagnosis until recruitment (‘historical’ data). Principal component analysis derived a factor, *g*, of general ability from seven cognitive tests. Linear regression models of follow-up *g* were adjusted for baseline *g* to represent 4 year cognitive change. ‘Accelerated late-life cognitive decline’ was defined as scoring in the lowest tertile of ‘4 year cognitive change’ regression scores. Analyses controlled for age and sex.

**Results:**

A baseline history of moderate/heavy smoking (≥10 pack-years) and a 1% increased historical HbA_1c_ (equivalent to an increase by 11 mmol/mol) predicted a 64% (OR 1.64; 95% CI 1.14, 2.34; *p* = 0.007) and 21% (OR 1.21; 95% CI 1.00, 1.45; *p* = 0.046) increased risk of accelerated cognitive decline, respectively. When treated as continuous measures, higher pack-years, historical HbA_1c_ and historical BP emerged as significant independent predictors of 4 year decline in *g* (standardised β range −0.07 to −0.14; all *p* ≤ 0.05).

**Conclusions/interpretation:**

Increased smoking and poorer glycaemic control (with relatively weaker findings for BP) during the life-course were independently associated with accelerated late-life cognitive decline. Where possible, evaluation is warranted of these risk factors as targets for intervention to reduce the burden of cognitive impairment in diabetes.

**Electronic supplementary material:**

The online version of this article (doi:10.1007/s00125-015-3581-0) contains peer-reviewed but unedited supplementary material, which is available to authorised users.

## Introduction

People with type 2 diabetes are at greater risk of cognitive impairment in later life than their non-diabetic peers [1]. With the prevalence of diabetes expected to reach 15% in the USA within the current decade [2], researchers have attempted to identify underlying pathophysiological mechanisms and potentially modifiable risk factors that may be responsible for the observation. For instance, based on competition of insulin with β-amyloid peptide for degradation and recent reports identifying the amyloid precursor amylin, which is co-secreted with insulin from the pancreas, in brains of patients with diabetes (but not in diabetes-free controls), increased concentrations of β-amyloid and amylin are candidate links between type 2 diabetes and cognitive impairment [3, 4]. The role of vascular disease has also received increasing attention of late due to an increased prevalence in type 2 diabetes and plausible biological mechanisms linking the two conditions. Amylin, for instance, is known to contribute to cardiovascular disease, so similar effects on the vascular system of the brain may be plausible [4]. Recent epidemiological evidence has also shown a direct association between cognitive decline and markers of symptomatic and asymptomatic vascular disease in people with type 2 diabetes [5]. These observations suggest a potential role for smoking, hypertension, adverse lipid profiles and hyperglycaemia, all of which are established risk factors for adverse cardiovascular outcomes, in the development of cognitive impairment in people with type 2 diabetes. If such a role is repeatedly demonstrated, then future clinical trials targeting these risk factors should consider inclusion of cognitive endpoints (e.g. to determine whether a change in the threshold for existing clinical intervention may be indicated).

In the general population, hypercholesterolaemia, hypertension, smoking and hyperglycaemia have each been associated with a poorer level of cognitive function [6–8]. Lower childhood intelligence, however, potentially leads to increased vascular risk, as well as to lower cognitive ability and greater cognitive decline in later life [9]. Therefore, studies with a prospective design and, preferably, with a measure of pre-morbid cognitive ability, are required to help determine the direction of relationships between vascular risk factors and cognitive decline. Compared with previous prospective studies in the general population that suggest a degree of complexity and do not always provide a consistent picture of vascular risk and cognitive impairment [10, 11], such studies in diabetic populations are much more scarce and often sub-optimal in design not least due to the neglect of consideration of pre-morbid ability [12]. For smoking in particular, this type of evidence was lacking until a recent analysis of the Fremantle Diabetes Study, which showed that midlife smoking predicted an increased risk of future cognitive impairment [13].

Using data from a large prospective cohort of older adults with type 2 diabetes, the Edinburgh Type 2 Diabetes Study (ET2DS), we tested the associations of serum cholesterol, BP, glycaemic control and smoking with late-life cognitive decline. Given known strong correlations among vascular risk factors themselves, it was important to evaluate the relative independence of their contributions in our cohort. Our analyses had the additional advantage of using ‘historical’ data on BP and HbA_1c_ covering the time between diabetes diagnosis and attendance at the clinic.

## Methods

### Study population

The baseline clinic of the ET2DS was attended by 1,066 community-dwelling adults aged 60–75 years [14], of whom 831 returned 4 years later. Ethical approval was obtained from the Lothian Medical Research Ethical Committee. Examinations complied with the Declaration of Helsinki and participants gave full written consent. Details of study recruitment and examination have been described previously [15].

### Baseline clinical examination and historical data

Data obtained at baseline (‘clinic data’) and from a clinical management database between diabetes diagnosis and attendance at the baseline clinic (‘historical data’) were used. At baseline, blood samples were taken following an overnight fast to measure HbA_1c_, plasma glucose, serum LDL-cholesterol and HDL-cholesterol. The ratio of HDL-cholesterol / total cholesterol was used (‘cholesterol’) in subsequent analysis as such ratios may be preferable to non-ratio measures in cardiovascular risk prediction [16].

Questionnaires administered at baseline obtained data on education, smoking and medical history. Medical history data, together with Scottish Morbidity Record Data, responses on the World Health Organization chest pain questionnaire and 12-lead ECG data, identified angina, transient ischaemic attack, stroke and myocardial infarction [14]. Hypertension was defined as systolic BP ≥ 140 mmHg and/or diastolic BP ≥ 85 mmHg at the clinic visit and/or when participants self-reported taking medication to lower BP. Hypercholesterolaemia was defined as plasma total cholesterol ≥ 5 mmol/l and/or when participants self-reported taking medication to lower blood lipids.

Questionnaire items ascertained current and previous smoking, including years since cessation and number of cigarettes, cigars or ounces of tobacco smoked per day. Each self-reported cigar smoked was converted to equal four cigarettes; each 20 cigarettes were converted to equal one pack. The number of packs was multiplied by the number of years participants had smoked to obtain ‘pack-years’ (1 pack-year is equivalent to 20 cigarettes/day for 1 year). For pipe smoking, 7,300 g of tobacco were equivalent to 1 pack-year. The measure of pack-years was additionally categorised into participants who had never smoked or had a history of light smoking (<10 pack-years) and those with a history of moderate to heavy smoking (≥10 pack-years).

Historical data from the Lothian Diabetes Register between 1988 and 2007 (the year of study recruitment) were used to obtain time-weighted mean measures of systolic and diastolic BP (‘historical systolic BP’; ‘historical diastolic BP’) and HbA_1c_ (‘historical HbA_1c_’). Data from between one reading and 65 readings (median 19 readings, interquartile range 14–25 readings) were available for each individual and were used to calculate the time-weighted mean. Assessments captured by the register spanned between 0 years (single assessment) and 19.8 years (median 10.4 years, interquartile range 7.3–13.6 years). ‘Poor glycaemic control’ was defined as a historical HbA_1c_ > 7% (>53 mmol/mol). ‘Poor BP control’ was defined as historical systolic BP ≥ 140 mmHg and/or historical diastolic BP ≥ 85 mmHg. Clinical characteristics of the study population are shown in Table 1.

**Table 1:** Baseline demographics and clinical characteristics of attendees of the year 4 follow-up

Characteristic/demographic	Total sample		Men		Women		*p* value^a^
	*N*	Mean ± SD, median (interquartile range) or *n* (%)	*N*	Mean ± SD, median (interquartile range) or *n* (%)	*N*	Mean ± SD, median (interquartile range) or *n* (%)	
Age (years)	831	67.7 ± 4.2	430	67.8 ± 4.1	401	67.6 ± 4.3	0.569
Duration of diabetes (years)	824	6 (3–11)	425	6 (4–11)	399	6 (3–10)	0.236
Insulin ± oral glucose-lowering treatment	830	139 (16.7)	429	66 (15.4)	399	73 (18.2)	0.756
Oral glucose-lowering treatment alone	830	526 (63.4)	429	276 (64.3)	399	250 (62.4)	
Diet alone as glucose-lowering treatment	830	165 (19.9)	429	87 (20.3)	399	78 (19.5)	
Lipid-lowering treatment	831	719 (86.5)	430	368 (85.6)	401	351 (87.5)	0.411
Hypercholesterolaemia	831	770 (92.7)	430	394 (91.6)	401	376 (93.8)	0.238
Total cholesterol (mmol/l)	826	4.3 ± 0.9	427	4.2 ± 0.8	399	4.5 ± 0.9	<0.001
HDL/total cholesterol	826	0.30 ± 0.09	427	0.29 ± 0.08	399	0.32 ± 0.08	<0.001
Historical systolic BP (mmHg)	825	139 ± 11	425	139 ± 10	400	139 ± 11	0.509
Historical diastolic BP (mmHg)	825	79 ± 6	425	79 ± 6	400	79 ± 6	0.189
Clinic systolic BP (mmHg)	829	133 ± 16	430	133 ± 15	399	132 ± 17	0.575
Clinic diastolic BP (mmHg)	829	69 ± 9	430	70 ± 9	399	67 ± 8.7	<0.001
Antihypertensive treatment	826	684 (82.8)	426	359 (84.3)	400	325 (81.3)	0.250
Hypertension	831	729 (87.7)	430	384 (89.3)	401	345 (86.0)	0.151
Poor blood pressure control	825	389 (47.2)	425	193 (45.4)	401	196 (49.0)	0.302
Pack-years	803	6 (0–30)	410	17 (0–40)	393	0 (0–20)	<0.001
Never smoked/history of light smoking	803	425 (52.9)	420	166 (40.5)	393	259 (65.9)	<0.001
History of moderate/heavy smoking	803	378 (47.1)	420	422 (59.5)	393	134 (34.1)	
Historical HbA_1c_ (%)	825	7.4 ± 0.9	425	7.3 ± 0.9	400	7.5 ± 0.9	0.061
Historical HbA_1c_ (mmol/mol)	825	57 ± 10	425	56 ± 10	400	59 ± 10	
Clinic HbA_1c_ (%)	804	7.4 ± 1.1	415	7.4 ± 1.2	389	7.4 ± 1.1	0.386
Clinic HbA_1c_ (mmol/mol)	804	57 ± 12	415	57 ± 13	389	57 ± 12	
Clinic plasma glucose (mmol/l)	821	7.2 (6.2–8.4)	424	7.3 (6.3–8.5)	397	7.1 (6.0–8.2)	0.059
Poor glycaemic control	825	560 (67.9)	425	279 (65.6)	400	281 (70.3)	0.157
History of severe hypoglycaemia	816	77 (9.4)	425	33 (7.8)	391	44 (11.3)	0.089
Diabetic retinopathy	819	266 (32.5)	424	155 (36.6)	395	111 (28.1)	0.010
Waist–hip ratio	828	0.97 ± 0.08	429	1.00 ± 0.06	399	0.93 ± 0.07	<0.001
Carotid intima-media thickness (mm)	775	1.00 ± 0.17	399	1.03 ± 0.18	376	0.96 ± 0.16	<0.001
Myocardial infarction	831	111 (13.4)	430	83 (19.3)	401	28 (7.0)	<0.001
Angina	831	222 (26.7)	430	137 (31.9)	401	85 (21.2)	0.001
Stroke	831	44 (5.3)	430	32 (7.4)	401	12 (3.0)	0.004
Transient ischaemic attack	831	27 (3.2)	430	16 (3.7)	401	11 (2.7)	0.427

Total *N* = 831

Poor glycaemic control was defined as historical HbA_1c_ >7% (>53 mmol/mol). Poor blood pressure control was defined as historical systolic BP ≥140 mmHg and/or historical diastolic BP ≥85 mmHg. Hypertension was defined as systolic BP ≥140 mmHg and/or diastolic BP ≥85 mmHg at the clinic visit and/or self-reported medication prescribed by a doctor to lower blood pressure. Hypercholesterolaemia was defined as plasma total cholesterol ≥5 mmol/l and/or when a participant self-reported medication prescribed by a doctor to lower blood lipid level. Never smoked/history of light smoking was defined as <10 pack-years. History of moderate/heavy smoking was defined as ≥10 pack-years. Carotid intima-media thickness was measured at year 1 follow-up. Diabetic retinopathy was defined as mild or moderate/severe retinopathy on seven-field retinal photographs. History of severe hypoglycaemia was defined as self-reported history of at least one episode of hypoglycaemia requiring assistance by another person (for details, see [32])

^a^ *p* value for sex difference in *χ* ^2^ tests or *t* tests

### Cognitive assessment

At baseline and at year 4, seven age-sensitive tests of cognitive function were administered. Executive function was measured by the Trail-Making Test-B (TMT-B) and the Borkowski Verbal Fluency Test (BVFT). The Digit Symbol Coding (DSC) subtest of the Wechsler Adult Intelligence Scale, third edition (WAIS-III) ascertained processing speed [17]. The Matrix Reasoning (MR) subtest of the WAIS-III measured non-verbal reasoning and the Letter–Number Sequencing (LNS) subtest measured working memory. The Logical Memory (LM) and Faces subtests of the Wechsler Memory Scale, third edition, assessed verbal and non-verbal declarative memory, respectively [18]. Scores on the combined junior and senior synonyms of the Mill Hill Vocabulary Scale (MHVS) [19], which have been shown to correlate with scores on other vocabulary-based tests [20], estimated pre-morbid cognitive ability. Vocabulary-based tests are used in this function on the grounds that vocabulary is part of ‘crystallised’ ability and hence is relatively immune to age-related cognitive decline [21]. The criteria used to identify participants with possible dementia by year 4 have been described previously [5].

### Statistical analyses

Distribution was approximately normal for all measured variables; for TMTB and clinic plasma glucose, normal distribution was achieved following transformation to their natural logarithms. Normal distribution for pack-years was achieved using square root transformation. Missing data on cognitive ability tests other than Mill Hill Vocabulary (0.6–1.7% at baseline; 0.8–4.7% at year 4) were imputed, as described in detail previously [5]. Since people who perform well on one cognitive test tend to perform well on another, data reduction techniques may be applied to batteries of cognitive tests with complete data on all individual tests to obtain a factor *g* of general ability [22]. The use of *g* is advantageous, because it is relatively unaffected by measurement error. Here, components with eigenvalues >1 were extracted in principal component analysis. All seven cognitive tests (those other than Mill Hill Vocabulary) loaded on a single component, accounting for 44.74% and 47.44% of variance at baseline and at year 4, respectively. Factor loadings ranged between 0.47 (Faces) and 0.80 (TMTB) at baseline and between 0.51 (Faces) and 0.81 (TMTB) at year 4 [5].

Linear regression analyses regressed cognitive function at year 4 and cognitive change between baseline and year 4 on each of the vascular risk factors (see Table 2 for analyses of *g*; see Electronic supplementary material [ESM] Table 1 for analyses of individual cognitive tests and ‘estimated lifetime cognitive change’, determined by adjustment of year 4 cognitive test scores for Mill-Hill Vocabulary). Four year cognitive change was represented by adjustment of year 4 scores for baseline scores (the procedure to ensure that baseline and year 4 *g* were standardised on the same sample has been described previously [5]). This method may be preferable to raw change scores because it is less dependent on individual differences in initial cognitive status [23].

**Table 2:** Clinical predictors of global cognitive performance (*g*) at year 4 and of 4 year change in *g* adjusted for MHVS and other covariates

Predictor	β adjusted for age and sex	4 year cognitive change		
		Adjusted for age, sex, baseline *g*	Adjusted for age, sex, baseline *g*, covariates	Adjusted for age, sex, baseline *g*, covariates, MHVS
Cholesterol	0.08 (0.017)	0.01 (0.823)	0.00 (0.938)	−0.01 (0.981)
Hypercholesterolaemia	0.02 (0.524)	0.02 (0.630)	0.02 (0.543)	0.02 (0.601)
Historical systolic BP	0.00 (0.992)	−0.07 (0.052)	−0.07 (0.067)	−0.07 (0.051)
Historical diastolic BP	0.05 (0.149)	−0.01 (0.750)	−0.02 (0.602)	−0.03 (0.375)
Poor BP control	0.01 (0.815)	−0.01 (0.810)	0.00 (0.931)	−0.01 (0.711)
Hypertension	0.01 (0.740)	0.00 (0.968)	0.01 (0.764)	0.03 (0.470)
Pack-years	−0.15 (<0.001)	−0.15 (<0.001)	−0.14 (<0.001)	−0.12 (0.002)
Historical HbA_1c_	−0.11 (0.001)	−0.11 (0.001)	−0.10 (0.005)	−0.10 (0.004)
Poor glycaemic control	−0.03 (0.337)	−0.05 (0.149)	−0.05 (0.144)	−0.04 (0.264)
Clinic plasma glucose	0.04 (0.296)	0.03 (0.350)	0.04 (0.216)	0.04 (0.296)

Data are shown as standardised β coefficients (*p* values). *N* = 778–823

Results are from multiple linear regression models performed separately for each risk factor. Outcome variable is *g* at year 4. Adjustment of year 4 scores for baseline scores represented 4 year change in cognitive performance. Pack-years are square root transformed. Clinic plasma glucose was transformed to its natural logarithm. Hypertension was defined as systolic BP ≥140 mmHg and/or diastolic BP ≥85 mmHg and/or self-reported medication prescribed by a doctor to lower BP. Hypercholesterolaemia was defined as plasma total cholesterol ≥5 mmol/l and/or self-reported medication prescribed by a doctor to lower blood lipids level. Poor glycaemic control was defined as historical HbA_1c_ >7% (>53 mmol/mol). Poor BP control was defined as historical systolic BP ≥140 mmHg and/or historical diastolic BP ≥85 mmHg. Covariates are baseline myocardial infarction, transient ischaemic attack, stroke, angina, duration of diabetes. MHVS, Mill Hill Vocabulary Scale

Regression analyses of cognitive function at year 4 were adjusted for age and sex; those of 4 year cognitive change were adjusted for baseline cognitive scores, age and sex, before myocardial infarction, angina, transient ischaemic attack, stroke (‘vascular disease’) and duration of diabetes, and Mill-Hill Vocabulary were controlled for in two separate steps. For *g*, analyses that survived this adjustment were additionally controlled for mode of glucose-lowering treatment. Finally, pack-years, historical systolic BP, historical HbA_1c_ and cholesterol were selected a priori (with the aim of avoiding multicollinearity) to be entered as predictors in a single model to ascertain the relative independence of associations. Additional categorisation of cognitive outcomes allowed the calculation of ORs for ‘poor’ cognitive function (scoring in lowest vs highest tertile of follow-up *g*) and ‘accelerated’ 4 year cognitive decline (lowest vs highest tertile of follow-up *g* adjusted for baseline *g*) using logistic regression. All linear and logistic regression analyses of *g* were repeated separately with stratification by sex (see Table 3 and ESM Table 2), on the basis of original rather than imputed data, and with exclusion of cases with possible dementia (*n* = 4); none of the findings were altered in these latter two steps unless noted in the text.

**Table 3:** Incremental odds of poor cognitive performance at year 4 and of accelerated 4 year cognitive decline according to preceding vascular risk factors

Risk factor	Poor cognitive function at year 4^a^	*p* value	Accelerated cognitive decline^b^	*p* value
Entire sample (max *N* = 831)				
Historical systolic BP	1.00 (0.98, 1.01)	0.635	1.01 (0.99, 1.03)	0.228
History of moderate/heavy vs never smoked/history of light smoking	2.01 (1.38, 2.92)	<0.001	1.64 (1.14, 2.34)	0.007
Pack-years	1.01 (1.00, 1.02)	0.001	1.01 (1.00, 1.01)	0.013
Historical HbA_1c_	1.24 (1.02, 1.49)	0.029	1.21 (1.00, 1.45)	0.046
Men (max *N* = 430)				
Historical systolic BP	1.00 (0.97, 1.02)	0.651	1.02 (1.00, 1.05)	0.053
History of moderate/heavy vs never smoked/history of light smoking	2.09 (1.25, 3.52)	0.005	1.61 (0.97, 2.66)	0.065
Pack-years	1.01 (1.00, 1.02)	0.036	1.01 (1.00, 1.02)	0.154
Historical HbA_1c_	1.34 (1.02, 1.77)	0.035	0.95 (0.73, 1.24)	0.706
Women (max *N* = 401)				
Historical systolic BP	1.00 (0.98, 1.02)	0.932	1.00 (0.98, 1.02)	0.802
History of moderate/heavy vs never smoked/history of light smoking	1.83 (1.06, 3.17)	0.030	1.66 (0.99, 2.77)	0.054
Pack-years	1.02 (1.01, 1.04)	0.003	1.02 (1.00, 1.03)	0.023
Historical HbA_1c_	1.14 (0.88, 1.45)	0.326	1.50 (1.15, 1.96)	0.003

Data are ORs (95% CIs) comparing incremental odds of scoring in the lowest tertile of the respective distributions vs the highest tertile

Analyses are multiple logistic regression analyses that were performed separately for each risk factor. Never smoked/history of light smoking was defined as <10 pack-years. History of moderate/heavy smoking was defined as ≥10 pack-years. Pack-years was untransformed in these analyses. Outcome variable is *g* at year 4. Analyses for entire sample was adjusted for age, sex and in sex-stratified analyses for age. Cut-points for follow-up *g*: lowest tertile < −0.43; medium tertile −0.43 to 0.48; highest tertile >0.48. Cut-points for follow-up *g* adjusted for baseline *g*: lowest tertile < −0.39; medium tertile −0.39 to 0.44; highest tertile >0.44

^a^Model of reduced cognitive function at year 4 was defined as scoring in the lowest tertile of *g* at year 4

^b^Model of accelerated 4 year cognitive decline was defined as scoring in the lowest tertile of year 4 *g* adjusted for baseline *g*

## Results

### Cohort characteristics

Eight hundred and thirty-one participants (51.7% male) of the ET2DS attended for follow-up cognitive testing. Clinical and cognitive differences between attenders and non-attenders have been described previously [5]. Baseline clinical and demographic characteristics of attendees (forming the study population for this analysis) are shown in Table 1. Mean age at baseline was 67.7 years, with median time since diabetes diagnosis of 6 years. Almost all participants suffered from hypercholesterolaemia (92.7%) and/or hypertension (87.7%); a majority had poor glycaemic control (67.9%) and around half (47.2%) had poor blood pressure control, according to our pre-specified criteria, respectively.

### Vascular risk and cognition in total sample

#### Blood pressure

Mill Hill Vocabulary was unrelated to historical diastolic (*p* = 0.076) or systolic BP (*p* = 0.701). Higher historical systolic BP was marginally associated with a steeper 4 year decline in *g* (Table 2), mainly due to an association with reasoning abilities (Matrix Reasoning) (ESM Table 1). Adjusting the association with 4 year decline in *g* for vascular disease, disease duration and Mill Hill Vocabulary did not alter the results in terms of effect size or statistical significance, and adjusting for glucose-lowering treatment led to statistical significance (standardised *β* −0.07; *p* = 0.047). Upon exclusion of individuals with possible dementia, the finding for *g* (standardised *β* −0.06; *p* = 0.069) was attenuated. When analyses were repeated with non-imputed rather than imputed data, the marginal association with 4 year decline in *g* lost statistical significance (−0.06; *p* = 0.125). BP was unrelated to risk of poor cognitive outcome (Table 3).

#### Cholesterol

People with higher cholesterol levels tended to have higher Mill Hill Vocabulary (*r* = 0.07; *p* = 0.054). A statistically significant association of higher cholesterol level with lower *g* did not survive adjustment for Mill Hill Vocabulary (ESM Table 1) or for baseline *g* (Table 2), and logistic regression analyses showed that cholesterol was unrelated to risk of poor cognitive outcome (data not shown).

#### Smoking

Pack-years was unrelated to Mill Hill Vocabulary (*p* = 0.157), but was associated with lower *g* at baseline and with accelerated decline in *g* (Table 2). Neither inclusion of vascular disease, disease duration and Mill Hill Vocabulary (Table 2) nor of glucose-lowering treatment (data not shown) altered the results. In addition to *g*, pack-years was associated with decline on all of the seven individual tests (ESM Table 1). Overall, individuals with a history of moderate to heavy smoking had a 64% increased odds of accelerated 4 year cognitive decline after controlling for age and sex. To illustrate, independent of age and sex, each additional pack-year was associated with 1% increased odds of accelerated 4 year decline (Table 3).

#### Historical HbA_1c_

Historical HbA_1c_ was unrelated to Mill Hill Vocabulary (*p* = 0.311), but was associated with lower *g* and with accelerated decline in *g* (Table 2). It was also significantly associated with decline on all tests except verbal memory (Logical Memory) and processing speed (Digit Symbol Coding; ESM Table 1). Adjustment for vascular disease, disease duration and Mill Hill Vocabulary (Table 2; ESM Table 1), or for glucose-lowering treatment (data not shown), did not alter the results. Each percentage (11 mmol/mol) increase in historical HbA_1c_ was associated with a 21% increased odds of accelerated 4 year decline when age and sex were controlled for (Table 3).

### Relative independence of vascular risk associations with cognition in total sample

Historical HbA_1c_, historical systolic BP, cholesterol and pack-years were entered in a single linear regression model controlling for age and sex. Significant contributors to 4 year change in *g* were pack-years (−0.14; *p* < 0.001), historical HbA_1c_ (−0.10; *p* = 0.006) and historical systolic BP (−0.07; *p* = 0.052). The addition of vascular disease, duration of diabetes and Mill-Hill Vocabulary into the model did not alter the results: pack-years (−0.11; *p* = 0.004), historical HbA_1c_ (−0.09; *p* = 0.014) and historical systolic BP (−0.07; *p* = 0.050) were each associated with a steeper 4 year decline in *g*. Cholesterol was not included in these models (both *p* > 0.10).

### Vascular risk and cognition in stratified analyses

Subgroup analysis by glucose-lowering treatment mode showed that findings on historical HbA_1c_ in the total sample were driven by insulin-treated patients: for this treatment group, effect sizes in linear regression analyses appeared to be particularly strong (fully adjusted model for 4 year decline in *g*, standardised *β* −0.22; *p* < 0.001). Findings on historical HbA_1c_ also appeared to be driven by female sex, whereas those for historical systolic BP were restricted to the subsample of men (ESM Table 2). When historical HbA_1c_, historical systolic BP, cholesterol and pack-years were entered in a single model controlling for age, vascular disease and duration of diabetes, significant predictors of a steeper 4 year decline were pack-years (−0.13; *p* = 0.011) and historical systolic BP (−0.12; *p* = 0.018) for the subsample of men (*p* > 0.05 for cholesterol and historical HbA_1c_, respectively), and pack-years (−0.13; *p* = 0.012) and historical HbA_1c_ (−0.16; *p* = 0.003) for women (*p* > 0.05 for cholesterol and historical systolic BP, respectively). These sex-specific patterns of associations were supported by findings from logistic regression analyses (Table 3).

## Discussion

Smoking history, long-term exposure to raised BP and poorer glycaemic control were independently associated with an accelerated late-life cognitive decline short of dementia. Overall effect sizes were modest but, compared with the findings on BP, those for a measure of mean HbA_1c_ collected over a time period of up to 20 years prior to cognitive testing and for lifetime smoking history appeared to be more robust across the total sample. Independent of age and sex, a history of moderate to heavy smoking and each percentage increase in HbA_1c_ (an increase that is equivalent to 11 mmol/mol) was associated with a 64% and 21% increased risk of an accelerated cognitive decline during 4 year follow-up, respectively. Cholesterol was associated only cross-sectionally with cognition. While our findings are not directly transferable to a clinical setting (since our definition of accelerated decline was arbitrary and sample-specific), they should serve to increase awareness of cognitive impairment and the potential importance of treatment of vascular risk factors in patients with type 2 diabetes.

To date, the relationships between cardiovascular risk factors and cognition have only infrequently been studied in people with diabetes. The present study has extended earlier evidence of cross-sectional associations of hypertension [24, 25] and poorer glycaemic control [26, 27] with reduced cognitive function in type 2 diabetes by demonstrating prospective associations with accelerated late-life cognitive decline. The association of smoking history with cognitive decline supports recent findings from the Fremantle Diabetes Study, in which midlife smoking was associated with the risk of cognitive impairment in later life [13]. Findings on glycaemic control in the present cohort are also consistent with a recent analysis of the Atherosclerosis Risk in Communities (ARIC) study, which reported that people with diabetes in midlife declined cognitively at faster rates during a 20 year follow-up period compared with diabetes-free individuals [28] and which additionally suggested a role for severity of hyperglycaemia [28]. In our study, the corresponding association of historical blood glucose levels with cognitive decline in people with diabetes in later life was particularly evident for tests of executive function, which again is consistent with findings from the ARIC study [28] and with reports of vulnerability of that domain in people with diabetes [29]. The suggestion of stronger findings for glycaemic control in insulin-treated patients in the present study may be due to the advanced disease stage and comorbidity in these patients and, in women, merits further investigation.

Many previous studies on a similar range of risk factors have investigated single factors, despite complex relationships among them. The present study makes an important advance by showing that associations of each of the risk factors identified as being predictive of cognitive decline were independent of one another. Although observational findings do not correspond to causality, it is possible that each factor is associated with cognition in separate pathophysiological pathways. Their modifiable nature suggests the potential for each risk factor as a separate target for intervention to reduce patients’ risk of cognitive impairment. Observational data from studies of people with type 2 diabetes appear to support this possibility (e.g. with associations of antihypertensive treatment with reduced risk of dementia as an endpoint of cognitive decline [30]) but current evidence from trials is limited and inconclusive. In the Memory in Diabetes study of the Action to Control Cardiovascular Risk in Diabetes (ACCORD-MIND) trial, a trend was observed for decelerated cognitive decline in the treatment arm that was subjected to intensified glucose-lowering treatment [31]. However, some evidence suggests that hypoglycaemia (a side effect of more intensive therapy) may increase the rate of cognitive decline [32–35], thereby potentially counteracting the beneficial effects of improved glycaemic control. Future trials comparing glucose-lowering agents that cause different degrees of hypoglycaemia may help to clarify this. Trials on smoking cessation are complicated by confounding and are scarce even in the general population, with a single study showing decelerated 2 year cognitive decline in people who successfully quit smoking compared with those who were unsuccessful [36]. A Cochrane review of randomised trials in the general population concluded that antihypertensive therapy was of no benefit in slowing cognitive decline [37] and in the ACCORD-MIND study, intensive lowering of BP in people with type 2 diabetes had no effect on cognitive decline [38].

The present findings are consistent with those of a number of studies that have reported links of higher midlife or late-life cholesterol, or of lower HDL-cholesterol, with lower late-life cognitive function or with a steeper cognitive decline in people with diabetes [11, 39, 40]. However, these studies did not adjust for pre-morbid ability. Here, we found evidence for reverse causality: lower pre-morbid ability predisposed participants to a higher late-life level of cholesterol and to lower late-life cognitive function, and so confounding of previous cross-sectional (and potentially prospective) evidence is likely. In line with the present null findings for prospective associations of either cholesterol (without regard to lipid-lowering treatment) or of presence of hypercholesterolaemia (partly defined by use of lipid-lowering treatment), ACCORD-MIND revealed no effect of addition of fenofibrate to simvastatin therapy on 3 year cognitive decline [38].

The prospective design and extensive clinical and cognitive characterisation are clear strengths of the ET2DS. Adjustment for estimated peak pre-morbid ability helped to counteract potential reverse causality. The dropout of 22% over 4 years was relatively high but acceptable considering the diabetic status and age of the cohort. The associated survivor bias favouring healthier individuals to attend follow-up is likely to have led to an underestimate in effect sizes, so that associations may be stronger than reported here. Our overall results are further strengthened by the use of historical data. Despite data heterogeneity (historical data were available for between 1 and 65 readings), which may decrease the overall reliability of our findings, the approach substantially reduced the influence of measurement error compared with single measurement data. The calculation of a *g* factor allowed the analysis of decline in overall cognitive function that was independent of the specific test battery used to measure cognitive function [41]. A resulting neglect of test-specific variance [42] was partly offset by additional consideration of findings from individual cognitive tests. Multiple testing will have increased the risk of type I statistical error and subgroup analysis by sex will have reduced statistical power, limiting the reliability of all findings and of those stratified by sex in particular. Replication in other cohorts with type 2 diabetes is therefore essential. Readers should also note that the category of accelerated cognitive decline was specific to the current, predominantly dementia-free cohort, and should not be mistaken for the presence of dementia. Finally, we did not consider the role of treatment of vascular risk, and so potential moderation of risk factor associations with cognitive decline by treatment cannot be excluded. The contributions of insulin or risk factor trajectories between baseline and year 4 follow-up to findings were also not assessed; such analyses are currently planned as part of future follow-up waves of the ET2DS.

Should associations of long-term exposure to vascular risk with cognitive decline prove to be causal, the present findings suggest that interventions targeting a range of vascular risk factors (rather than single factors such as hyperglycaemia only) may be needed to help reduce the risk of cognitive impairment in people who either have diabetes or who go on to develop type 2 diabetes in older age. Our findings support recent evidence that late-life cognitive change may be determined over the course of decades prior to the age at which deficits typically occur [28].

Overall, we have provided strong observational evidence showing that smoking history, higher BP and poorer glycaemic control are independently associated with accelerated late-life cognitive decline in people with type 2 diabetes. Further research into the usefulness of each risk factor as a target for intervention to reduce age-related cognitive decline in this patient population is now warranted.

## Electronic supplementary material

ESM Table 1 (PDF 157 kb)

ESM Table 2 (PDF 91.4 kb)

## Abbreviations

ACCORD-MIND: Action to Control Cardiovascular Risk in Diabetes
ARIC: Atherosclerosis Risk in Communities
BVFT: Borkowski Verbal Fluency test
DSC: Digit Symbol Coding
ET2DS: Edinburgh Type 2 Diabetes Study
*g*: Factor of general cognitive ability
LM: Logical Memory
LNS: Letter–Number Sequencing
MR: Matrix Reasoning
TMT-B: Trail-Making Test-B
WAIS-III: Wechsler Adult Intelligence Scale, third edition
