# Supplementary material for: Cardiovascular risk factors and cognitive decline in older people with type 2 diabetes
Source: Diabetologia. 2015 Apr 7;58(7):1637–45. doi: 10.1007/s00125-015-3581-0 (PMC4473016; doi:10.1007/s00125-015-3581-0)
Supplement: Supplementary file 1 — (PDF 157 kb) [file 125_2015_3581_MOESM1_ESM.pdf]

ESM Table 1: Linear regression models of level of and change in follow-up *g* and cognitive test scores on vascular risk factors

		Four-year cognitive change			Estimated lifetime cognitive change	
	Age and sex adjusted beta	Beta adjusted for age, sex, baseline score	Beta adjusted for age, sex, baseline score, covariates	Beta adjusted for age, sex, baseline score, covariates, MHVS	Beta adjusted for age, sex, MHVS	Beta adjusted for age, sex, MHVS, covariates
G						
Cholesterol					0.04 (0.176)	0.04 (0.208)
Hypercholesterolaemia					0.04 (0.181)	0.04 (0.157)
Historical systolic BP					-0.01 (0.827)	0.00 (0.873)
Historical diastolic BP					0.01 (0.861)	-0.01 (0.853)
Poor BP control					-0.01 (0.813)	0.00 (0.987)
Hypertension					0.04 (0.124)	0.06 (0.040)
Pack-years					-0.10 (0.001)	-0.10 (0.001)
Historical HbA_{1c}					-0.10 (0.001)	-0.07 (0.015)
Poor glycaemic control					-0.03 (0.243)	-0.01 (0.748)
Clinic plasma glucose					0.03 (0.289)	0.05 (0.104)
Logical Memory						
Cholesterol	0.09 (0.014)	0.05 (0.059)	0.05 (0·080)	0.04 (0.161)	0.05 (0.121)	0.05 (0.130)
Hypercholesterolaemia	0.01 (0.873)	0.03 (0.247)	0.04 (0·146)	0.04 (0.170)	0.02 (0.631)	0.02 (0.620)
Historical systolic BP	0.00 (0.905)	0.00 (0.965)	0.00 (0·973)	0.00 (0.998)	0.00 (0.975)	0.01 (0.884)
Historical diastolic BP	0.07 (0.039)	0.07 (0.011)	0.06 (0·023)	0.05 (0.068)	0.04 (0.227)	0.04 (0.200)
Poor BP control	0.00 (0.943)	0.01 (0.728)	0.01 (0·754)	0.00 (0.931)	-0.01 (0.771)	-0.01 (0.883)
Hypertension	-0.02 (0.634)	-0.02 (0.580)	-0.01 (0·831)	0.00 (0.900)	0.01 (0.805)	0.01 (0.721)
Pack-years	-0.05 (0.193)	-0.05 (0.088)	-0.05 (0·121)	-0.03 (0.357)	-0.01 (0.756)	-0.01 (0.772)
Historical HbA_{1c}	0.02 (0.514)	0.00 (0.971)	0.01 (0·632)	0.03 (0.291)	0.04 (0.159)	0.05 (0.098)
Poor glycaemic control	0.03 (0.336)	-0.01 (0.704)	0.00 (0·910)	0.01 (0.745)	0.04 (0.242)	0.04 (0.177)
Clinic plasma glucose	0.05 (0.151)	0.02 (0.394)	0.03 (0·238)	0.03 (0.212)	0.05 (0.120)	0.06 (0.082)
Faces						
Cholesterol	0.05 (0.145)	0.04 (0.136)	0.04 (0.163)	0.03 (0.264)	0.03 (0.407)	0.03 (0.382)
Hypercholesterolaemia	-0.02 (0.653)	0.00 (0.890)	0.01 (0.862)	0.00 (0.888)	0.01 (0.731)	-0.01 (0.757)
Historical systolic BP	-0.03 (0.468)	-0.03 (0.250)	-0.03 (0.229)	-0.04 (0.196)	-0.03 (0.402)	-0.03 (0.377)
Historical diastolic BP	0.04 (0.259)	0.02 (0.536)	0.01 (0.727)	0.00 (0.931)	0.02 (0.562)	0.00 (0.903)
Poor BP control	-0.02 (0.511)	-0.02 (0.519)	-0.02 (0.445)	-0.03 (0.309)	-0.03 (0.341)	-0.04 (0.262)
Hypertension	0.00 (0.943)	0.03 (0.337)	0.03 (0.278)	0.04 (0.138)	0.03 (0.385)	0.04 (0.259)
Pack-years	-0.14 (<0.001)	-0.09 (0.003)	-0.08 (0.006)	-0.07 (0.013)	-0.12 (0.001)	-0.12 (0.001)

Historical HbA_{1c}	-0.10 (0.003)	-0.06 (0.030)	-0.06 (0.049)	-0.06 (0.056)	-0.09 (0.005)	-0.07 (0.036)
Poor glycaemic control	-0.07 (0.052)	-0.04 (0.138)	-0.04 (0.184)	-0.04 (0.177)	-0.07 (0.039)	-0.05 (0.143)
Clinic plasma glucose	-0.03 (0.375)	-0.01 (0.837)	0.00 (0.947)	-0.01 (0.784)	-0.03 (0.298)	-0.03 (0.433)
Letter-Number Sequencing						
Cholesterol	0.04 (0.314)	0.01 (0.637)	0.01 (0.686)	0.00 (0.912)	0.00 (0.956)	0.00 (0.987)
Hypercholesterolaemia	0.02 (0.482)	0.02 (0.606)	0.02 (0.421)	0.03 (0.283)	0.04 (0.235)	0.04 (0.183)
Historical systolic BP	0.01 (0.842)	-0.02 (0.603)	-0.01 (0.685)	-0.02 (0.611)	0.00 (0.915)	0.00 (0.924)
Historical diastolic BP	0.02 (0.607)	0.00 (0.947)	-0.01 (0.844)	-0.02 (0.434)	-0.02 (0.576)	-0.02 (0.470)
Poor BP control	0.03 (0.455)	0.01 (0.777)	0.01 (0.666)	0.01 (0.852)	0.01 (0.738)	0.02 (0.617)
Hypertension	0.02 (0.670)	0.02 (0.551)	0.03 (0.279)	0.05 (0.094)	0.04 (0.238)	0.05 (0.114)
Pack-years	-0.08 (0.040)	-0.07 (0.030)	-0.06 (0.056)	-0.04 (0.188)	-0.04 (0.242)	-0.03 (0.313)
Historical HbA_{1c}	-0.07 (0.040)	-0.07 (0.029)	-0.06 (0.068)	-0.05 (0.128)	-0.06 (0.072)	-0.04 (0.227)
Poor glycaemic control	0.00 (0.920)	-0.01 (0.709)	0.00 (0.985)	0.01 (0.788)	0.00 (0.996)	0.02 (0.591)
Clinic plasma glucose	0.02 (0.555)	0.03 (0.391)	0.03 (0.274)	0.03 (0.266)	0.02 (0.494)	0.03 (0.348)
Matrix Reasoning						
Cholesterol	0.05 (0.146)	0.00 (0.898))	-0.01 (0.846)	-0.01 (0.850)	0.02 (0.502)	0.02 (0.563)
Hypercholesterolaemia	0.01 (0.768)	-0.01 (0.590)	-0.01 (0.643)	-0.01 (0.776)	0.02 (0.534)	0.02 (0.443)
Historical systolic BP	-0.04 (0.247)	-0.06 (0.026)	-0.06 (0.018)	-0.06 (0.021)	-0.04 (0.183)	-0.04 (0.175)
Historical diastolic BP	-0.02 (0.591)	-0.05 (0.091)	-0.06 (0.026)	-0.07 (0.009)	-0.05 (0.107)	-0.06 (0.044)
Poor BP control	0.03 (0.408)	-0.03 (0.284)	-0.03 (0.278)	-0.03 (0.192)	-0.04 (0.224)	-0.04 (0.233)
Hypertension	-0.01 (0.760)	-0.04 (0.118)	-0.04 (0.182)	-0.02 (0.410)	0.01 (0.744)	0.02 (0.500)
Pack-years	-0.12 (0.001)	-0.06 (0.031)	-0.05 (0.055)	-0.04 (0.106)	-0.08 (0.021)	-0.07 (0.030)
Historical HbA_{1c}	-0.06 (0.077)	-0.05 (0.052)	-0.04 (0.208)	-0.03 (0.272)	-0.05 (0.113)	-0.03 (0.399)
Poor glycaemic control	-0.03 (0.372)	-0.04 (0.095)	-0.03 (0.255)	-0.03 (0.293)	-0.03 (0.278)	-0.01 (0.679)
Clinic plasma glucose	0.05 (0.143)	0.02 (0.379)	0.03 (0.266)	0.03 (0.250)	0.04 (0.158)	0.05 (0.098)
Trail-Making Test						
Cholesterol	-0.05 (0.176)	-0.02 (0.483)	-0.01 (0.625)	-0.01 (0.813)	-0.02 (0.544)	-0.02 (0.564)
Hypercholesterolaemia	-0.02 (0.587)	0.00 (0.910)	0.00 (0.879)	-0.01 (0.780)	-0.03 (0.340)	-0.03 (0.380)
Historical systolic BP	-0.01 (0.684)	0.01 (0.638)	0.00 (0.881)	0.01 (0.811)	-0.01 (0.740)	-0.03 (0.425)
Historical diastolic BP	-0.06 (0.099)	-0.02 (0.374)	-0.02 (0.453)	-0.01 (0.693)	-0.03 (0.398)	-0.02 (0.457)
Poor BP control	-0.03 (0.470)	-0.01 (0.839)	-0.01 (0.588)	-0.01 (0.799)	-0.01 (0.665)	-0.03 (0.391)
Hypertension	-0.06 (0.058)	-0.03 (0.280)	-0.03 (0.200)	-0.05 (0.082)	-0.09 (0.007)	-0.09 (0.003)
Pack-years	0.12 (0.001)	0.07 (0.013)	0.07 (0.019)	0.05 (0.063)	0.09 (0.012)	0.08 (0.013)
Historical HbA_{1c}	0.11 (0.002)	0.06 (0.026)	0.05 (0.047)	0.06 (0.032)	0.11 (0.001)	0.09 (0.008)
Poor glycaemic control	0.02 (0.523)	0.00 (0.973)	0.00 (0.867)	-0.01 (0.794)	0.02 (0.510)	0.01 (0.885)
Clinic plasma glucose	-0.05 (0.163)	-0.02 (0.385)	-0.03 (0.238)	-0.03 (0.316)	-0.04 (0.194)	-0.06 (0.080)
Verbal Fluency						

Cholesterol	0.10 (0.007)	0.00 (0.966)	0.00 (0.879)	-0.01 (0.821)	0.06 (0.063)	0.06 (0.057)
Hypercholesterolaemia	0.00 (0.959)	-0.01 (0.672)	-0.01 (0.712)	-0.01 (0.666)	0.02 (0.573)	0.01 (0.681)
Historical systolic BP	0.01 (0.784)	-0.02 (0.363)	-0.02 (0.423)	-0.01 (0.599)	0.01 (0.779)	0.02 (0.580)
Historical diastolic BP	0.04 (0.315)	0.00 (0.936)	0.00 (0.902)	-0.01 (0.806)	0.01 (0.868)	0.00 (0.984)
Poor BP control	0.01 (0.870)	0.00 (0.898)	0.00 (0.901)	0.00 (0.967)	0.00 (0.987)	0.00 (0.930)
Hypertension	-0.04 (0.318)	-0.03 (0.109)	-0.03 (0.149)	-0.03 (0.225)	-0.01 (0.728)	-0.01 (0.859)
Pack-years	-0.05 (0.183)	-0.05 (0.024)	-0.05 (0.025)	-0.04 (0.066)	-0.01 (0.716)	-0.02 (0.657)
Historical HbA_{1c}	-0.13 (<0.001)	-0.07 (<0.001)	-0.07 (0.001)	-0.07 (0.001)	-0.12 (<0.001)	-0.10 (0.003)
Poor glycaemic control	-0.06 (0.078)	-0.05 (0.015)	-0.05 (0.020)	-0.05 (0.017)	-0.06 (0.052)	-0.04 (0.228)
Clinic plasma glucose	0.00 (0.906)	-0.01 (0.756)	0.00 (0.978)	0.00 (0.892)	0.00 (0.923)	0.01 (0.778)
Digit Symbol Coding						
Cholesterol	0.05 (0.154)	0.01 (0.773)	0.00 (0.987)	0.00 (0.860)	0.02 (0.538)	0.01 (0.713)
Hypercholesterolaemia	0.04 (0.212)	0.03 (0.281)	0.03 (0.226)	0.03 (0.234)	0.05 (0.091)	0.06 (0.063)
Historical systolic BP	0.02 (0.667)	0.00 (0.881)	0.01 (0.621)	0.01 (0.619)	0.01 (0.749)	0.02 (0.452)
Historical diastolic BP	0.04 (0.298)	-0.01 (0.686)	-0.01 (0.580)	-0.02 (0.409)	0.01 (0.884)	-0.01 (0.806)
Poor BP control	0.01 (0.733)	0.01 (0.646)	0.02 (0.475)	0.01 (0.548)	0.00 (0.924)	0.01 (0.722)
Hypertension	0.01 (0.720)	0.03 (0.262)	0.03 (0.173)	0.04 (0.100)	0.03 (0.324)	0.05 (0.132)
Pack-years	-0.17 (<0.001)	-0.09 (0.001)	-0.09 (0.001)	-0.08 (0.001)	-0.14 (<0.001)	-0.14 (<0.001)
Historical HbA_{1c}	-0.10 (0.004)	-0.04 (0.097)	-0.04 (0.118)	-0.04 (0.106)	-0.09 (0.007)	-0.06 (0.052)
Poor glycaemic control	-0.03 (0.462)	0.01 (0.719)	0.01 (0.824)	0.00 (0.867)	-0.03 (0.419)	-0.01 (0.730)
Clinic plasma glucose	0.02 (0.639)	0.02 (0.524)	0.02 (0.339)	0.02 (0.445)	0.01 (0.691)	0.03 (0.332)

Data are shown as standardised β coefficients (*p* values). *N*=771 to 825. Results are from multiple linear regression models performed separately for each risk factor. Outcome variable is *g* at year 4. Adjustment of year 4 scores for baseline scores represented four-year change in cognitive performance. Cognitive test scores obtained at year 4 were adjusted for baseline MHVS to estimate the lifetime change in cognitive function. Pack-years are square root transformed. Clinic plasma glucose was transformed to its natural logarithm. MHVS, Mill Hill Vocabulary Scale. Hypertension was defined as systolic blood pressure ≥140mmHg and/or diastolic blood pressure ≥85mmHg and/or self-reported medication prescribed by a doctor to lower blood pressure. Hypercholesterolaemia was defined as plasma total cholesterol ≥ 5 mmol/L and/or self-reported medication prescribed by a doctor to lower blood lipids level. Poor glycaemic control was defined as historical HbA_{1c} >7% (>53 mmol/mol). Poor blood pressure control was defined as historical systolic blood pressure ≥140mmHg and/or historical diastolic blood pressure ≥85mmHg. Covariates are baseline myocardial infarction, transient ischaemic attack, stroke, angina, duration of diabetes.
