# Supplementary material for: Cardiovascular risk factors and cognitive decline in older people with type 2 diabetes
Source: Diabetologia. 2015 Apr 7;58(7):1637–45. doi: 10.1007/s00125-015-3581-0 (PMC4473016; doi:10.1007/s00125-015-3581-0)
Supplement: Supplementary file 2 — (PDF 91.4 kb) [file 125_2015_3581_MOESM2_ESM.pdf]

ESM Table 2: Results from multiple adjusted linear regression models of follow-up *g* and change in *g* on each of the vascular risk factors, for male and female subsamples

		Four-year cognitive change		
	Age and sex adjusted beta	Beta adjusted for age, sex, baseline *g*	Beta adjusted for age, sex, baseline *g*, covariates	Beta adjusted for age, sex, baseline *g*, covariates, MHVS
Men				
Cholesterol	0.08 (0.107)	-0.01 (0.860)	-0.01 (0.882)	-0.02 (0.767)
Hypercholesterolaemia	0.02 (0.695)	0.02 (0.650)	0.03 (0.598)	0.01 (0.817)
Historical systolic BP	-0.01 (0.916)	-0.13 (0.007)	-0.12 (0.013)	-0.15 (0.002)
Historical diastolic BP	0.07 (0.132)	-0.01 (0.803)	-0.02 (0.714)	-0.04 (0.436)
Poor BP control	0.02 (0.723)	-0.07 (0.166)	-0.05 (0.274)	-0.08 (0.085)
Hypertension	0.01 (0.795)	-0.03 (0.602)	-0.02 (0.690)	-0.01 (0.808)
Pack-years	-0.13 (0.008)	-0.14 (0.004)	-0.14 (0.005)	-0.11 (0.030)
Historical HbA_{1c}	-0.15 (0.001)	-0.04 (0.392)	-0.03 (0.451)	-0.03 (0.608)
Poor glycaemic control	-0.04 (0.395)	-0.02 (0.720)	-0.01 (0.909)	0.01 (0.861)
Clinic plasma glucose	0.03 (0.494)	0.09 (0.057)	0.10 (0.047)	0.10 (0.046)
Women				
Cholesterol	0.09 (0.072)	0.03 (0.603)	0.01 (0.927)	0.00 (0.990)
Hypercholesterolaemia	0.02 (0.625)	0.01 (0.845)	0.01 (0.773)	0.02 (0.694)
Historical systolic BP	0.00 (0.962)	0.00 (0.987)	0.00 (0.956)	0.01 (0.825)
Historical diastolic BP	0.02 (0.688)	0.00 (0.972)	-0.01 (0.864)	-0.01 (0.800)
Poor BP control	0.00 (0.946)	0.05 (0.279)	0.06 (0.277)	0.06 (0.231)
Hypertension	0.01 (0.886)	0.03 (0.603)	0.04 (0.451)	0.06 (0.231)
Pack-years	-0.16 (0.001)	-0.14 (0.006)	-0.12 (0.017)	-0.12 (0.025)
Historical HbA_{1c}	-0.08 (0.131)	-0.19 (<0.001)	-0.17 (0.002)	-0.17 (0.002)
Poor glycaemic control	-0.02 (0.658)	-0.09 (0.084)	-0.07 (0.174)	-0.08 (0.118)
Clinic plasma glucose	0.04 (0.410)	-0.03 (0.542)	-0.01 (0.784)	-0.02 (0.650)

Data are shown as standardised β coefficients (*p* values). *N*=389 to 424 for men; *N*= 387 to 399 for women. Results are from multiple linear regression models performed separately for each risk factor. Outcome variable is *g* at year 4. Pack-years are square root transformed. Clinic plasma glucose was transformed to its natural logarithm. MHVS, Mill-Hill Vocabulary Scale. Hypertension was defined as systolic blood pressure ≥140mmHg and/or diastolic blood pressure ≥85mmHg and/or self-reported medication prescribed by a doctor to lower blood pressure. Hypercholesterolaemia was defined as plasma total cholesterol ≥ 5 mmol/L and/or self-reported medication prescribed by a doctor to lower blood lipids level. Poor glycaemic control was defined as historical HbA_{1c} >7% (>53 mmol/mol). Poor blood pressure control was defined as historical systolic blood pressure ≥140mmHg and/or historical diastolic blood pressure ≥85mmHg.

Covariates are baseline myocardial infarction, transient ischaemic attack, stroke, angina, duration of diabetes.
